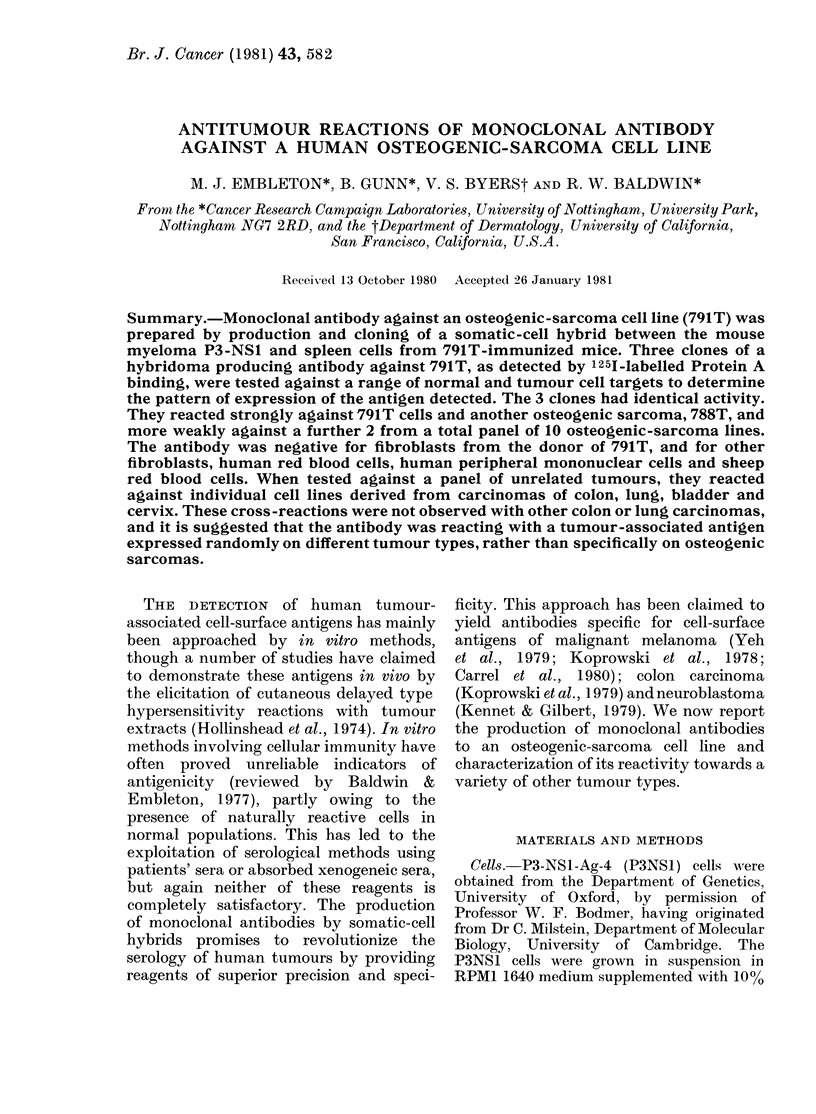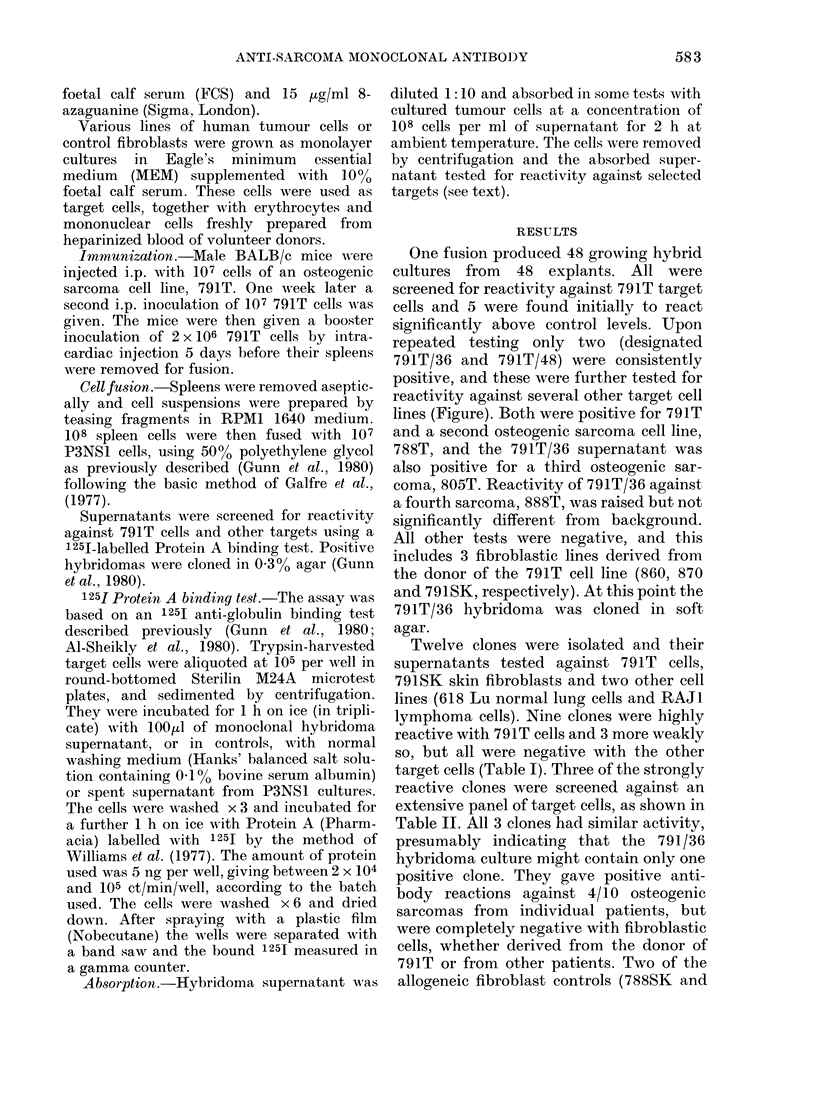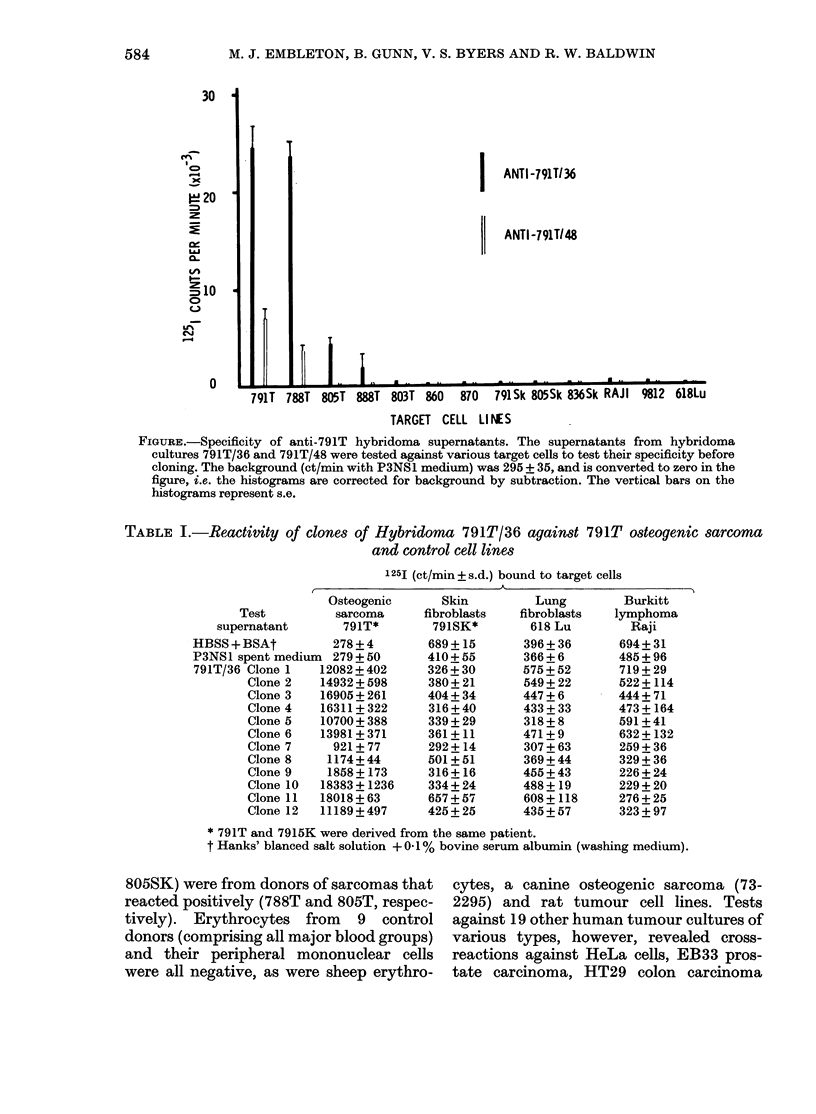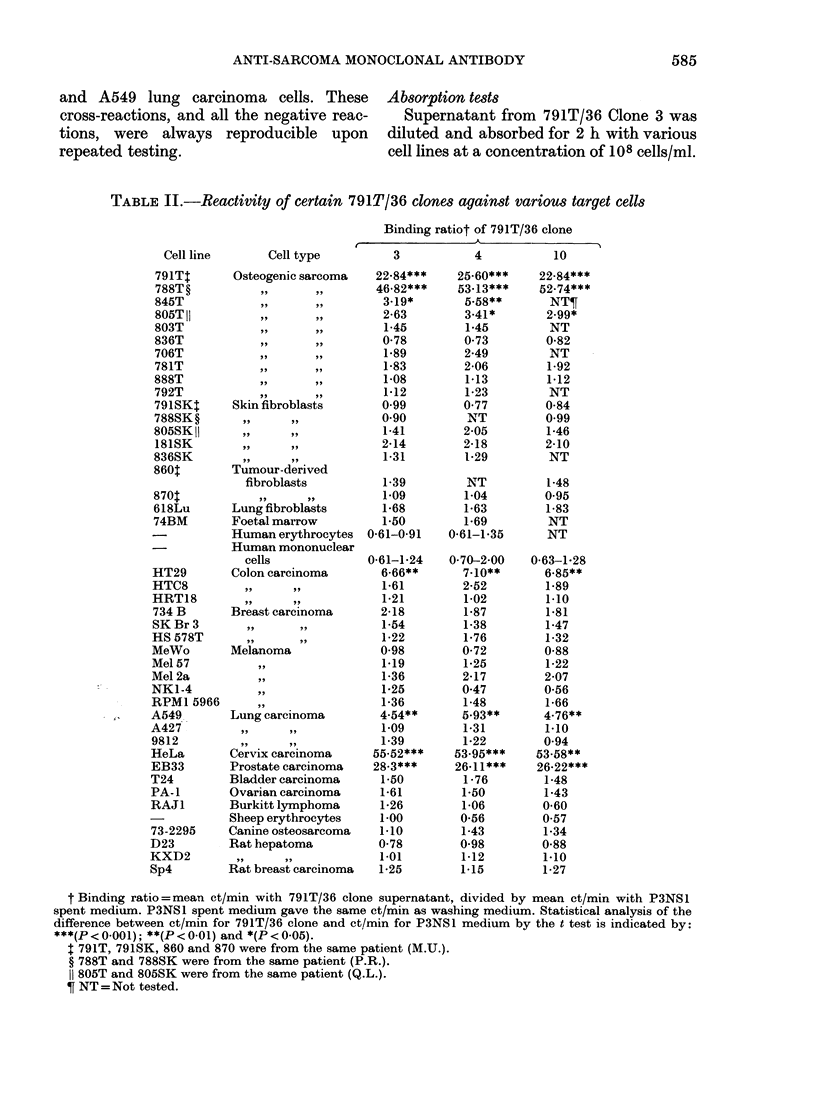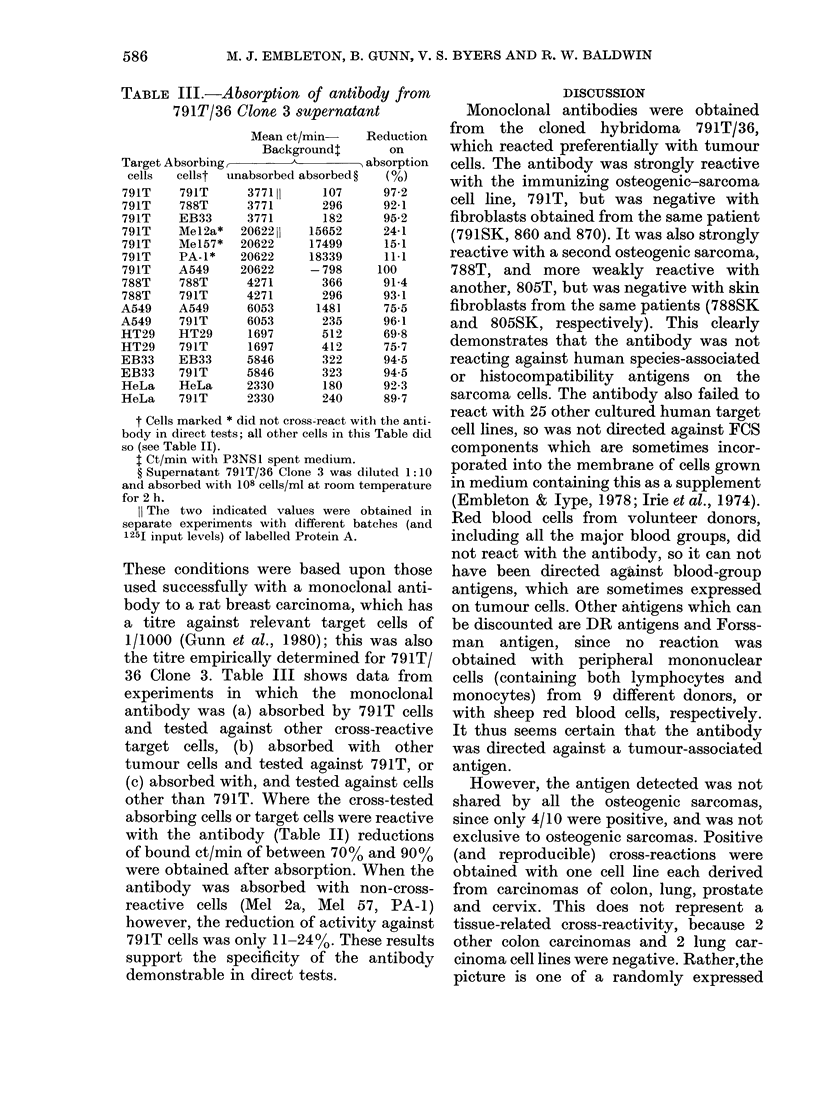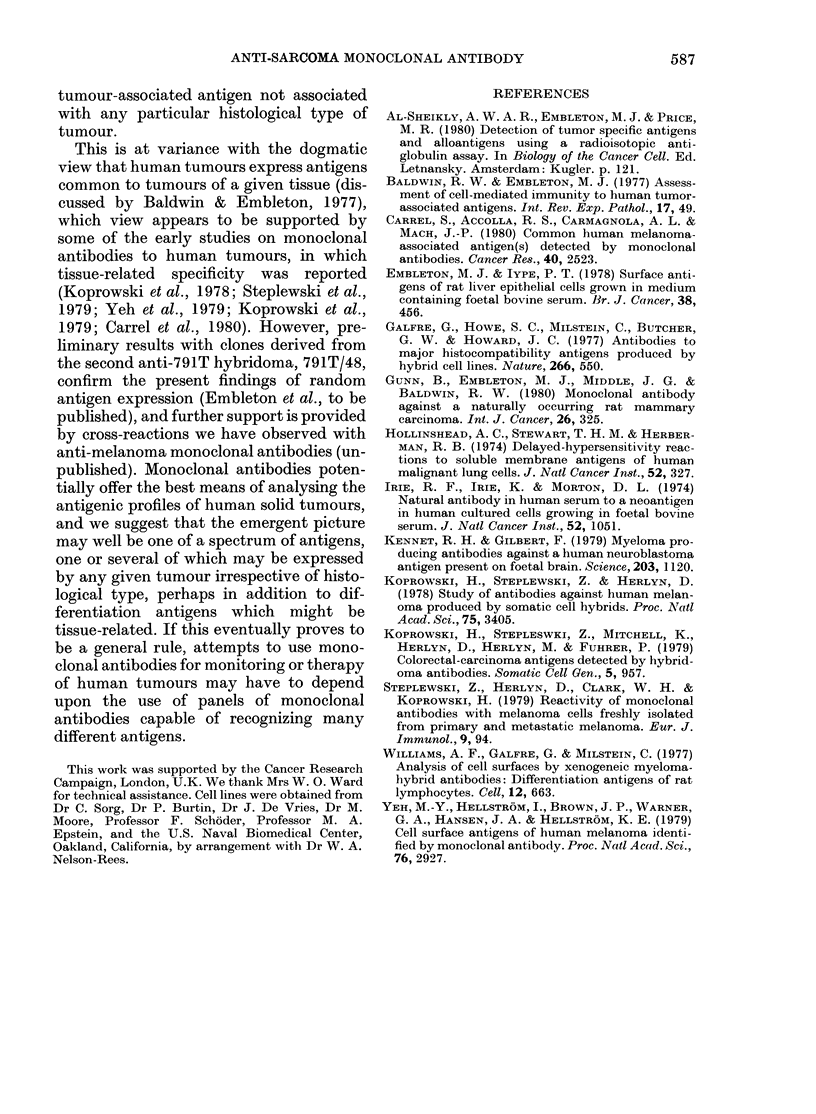# Antitumour reactions of monoclonal antibody against a human osteogenic-sarcoma cell line.

**DOI:** 10.1038/bjc.1981.87

**Published:** 1981-05

**Authors:** M. J. Embleton, B. Gunn, V. S. Byers, R. W. Baldwin

## Abstract

Monoclonal antibody against an osteogenic-sarcoma cell line (791T) was prepared by production and cloning of a somatic-cell hybrid between the mouse myeloma P3-NS1 and spleen cells from 791T-immunized mice. Three clones of hybridoma producing antibody against 791T, as detected by 125I-labelled Protein A binding, were tested against a range of normal and tumour cell targets to determine the pattern of expression of the antigen detected. The 3 clones had identical activity. They reacted strongly against 791T cells and another osteogenic sarcoma, 788T, and more weakly against a further 2 from a total panel of 10 osteogenic-sarcoma lines. The antibody was negative for fibroblasts from the donor of 791T, and for other fibroblasts, human red blood cells, human peripheral mononuclear cells and sheep red blood cells. When tested against a panel of unrelated tumours, they reacted against individual cell lines derived from carcinomas of colon, lung, bladder and cervix. These cross-reactions were not observed with other colon or lung carcinomas, and it is suggested that the antibody was reacting with a tumour-associated antigen expressed randomly on different tumour types, rather than specifically on osteogenic sarcomas.


*Br. J. Cancer* (1981) **43**, 582

### ANTITUMOUR REACTIONS OF MONOCLONAL ANTIBODY AGAINST A HUMAN OSTEOGENIC-SARCOMA CELL LINE

M. J. EMBLETON*, B. GUNN*, V. S. BYERS† AND R. W. BALDWIN*

*From the *Cancer Research Campaign Laboratories, University of Nottingham, University Park, Nottingham NG7 2RD, and the †Department of Dermatology, University of California, San Francisco, California, U.S.A.*

Received 13 October 1980 Accepted 26 January 1981

**Summary.—Monoclonal antibody against an osteogenic-sarcoma cell line (791T) was prepared by production and cloning of a somatic-cell hybrid between the mouse myeloma P3-NS1 and spleen cells from 791T-immunized mice. Three clones of a hybridoma producing antibody against 791T, as detected by $^{125}$I-labelled Protein A binding, were tested against a range of normal and tumour cell targets to determine the pattern of expression of the antigen detected. The 3 clones had identical activity. They reacted strongly against 791T cells and another osteogenic sarcoma, 788T, and more weakly against a further 2 from a total panel of 10 osteogenic-sarcoma lines. The antibody was negative for fibroblasts from the donor of 791T, and for other fibroblasts, human red blood cells, human peripheral mononuclear cells and sheep red blood cells. When tested against a panel of unrelated tumours, they reacted against individual cell lines derived from carcinomas of colon, lung, bladder and cervix. These cross-reactions were not observed with other colon or lung carcinomas, and it is suggested that the antibody was reacting with a tumour-associated antigen expressed randomly on different tumour types, rather than specifically on osteogenic sarcomas.**

THE DETECTION of human tumour-associated cell-surface antigens has mainly been approached by *in vitro* methods, though a number of studies have claimed to demonstrate these antigens *in vivo* by the elicitation of cutaneous delayed type hypersensitivity reactions with tumour extracts (Hollinshead *et al.*, 1974). *In vitro* methods involving cellular immunity have often proved unreliable indicators of antigenicity (reviewed by Baldwin & Embleton, 1977), partly owing to the presence of naturally reactive cells in normal populations. This has led to the exploitation of serological methods using patients' sera or absorbed xenogeneic sera, but again neither of these reagents is completely satisfactory. The production of monoclonal antibodies by somatic-cell hybrids promises to revolutionize the serology of human tumours by providing reagents of superior precision and specificity. This approach has been claimed to yield antibodies specific for cell-surface antigens of malignant melanoma (Yeh *et al.*, 1979; Koprowski *et al.*, 1978; Carrel *et al.*, 1980); colon carcinoma (Koprowski *et al.*, 1979) and neuroblastoma (Kennet & Gilbert, 1979). We now report the production of monoclonal antibodies to an osteogenic-sarcoma cell line and characterization of its reactivity towards a variety of other tumour types.

### MATERIALS AND METHODS

*Cells.*—P3-NS1-Ag-4 (P3NS1) cells were obtained from the Department of Genetics, University of Oxford, by permission of Professor W. F. Bodmer, having originated from Dr C. Milstein, Department of Molecular Biology, University of Cambridge. The P3NS1 cells were grown in suspension in RPMI 1640 medium supplemented with 10%

foetal calf serum (FCS) and 15 μg/ml 8-azaguanine (Sigma, London).

Various lines of human tumour cells or control fibroblasts were grown as monolayer cultures in Eagle's minimum essential medium (MEM) supplemented with 10% foetal calf serum. These cells were used as target cells, together with erythrocytes and mononuclear cells freshly prepared from heparinized blood of volunteer donors.

*Immunization*.—Male BALB/c mice were injected i.p. with $10^7$ cells of an osteogenic sarcoma cell line, 791T. One week later a second i.p. inoculation of $10^7$ 791T cells was given. The mice were then given a booster inoculation of $2 \times 10^6$ 791T cells by intracardiac injection 5 days before their spleens were removed for fusion.

*Cell fusion*.—Spleens were removed aseptically and cell suspensions were prepared by teasing fragments in RPM1 1640 medium. $10^8$ spleen cells were then fused with $10^7$ P3NS1 cells, using 50% polyethylene glycol as previously described (Gunn *et al*., 1980) following the basic method of Galfre *et al*., (1977).

Supernatants were screened for reactivity against 791T cells and other targets using a $^{125}$I-labelled Protein A binding test. Positive hybridomas were cloned in 0·3% agar (Gunn *et al*., 1980).

*$^{125}$I Protein A binding test*.—The assay was based on an $^{125}$I anti-globulin binding test described previously (Gunn *et al*., 1980; Al-Sheikly *et al*., 1980). Trypsin-harvested target cells were aliquoted at $10^5$ per well in round-bottomed Sterilin M24A microtest plates, and sedimented by centrifugation. They were incubated for 1 h on ice (in triplicate) with 100μl of monoclonal hybridoma supernatant, or in controls, with normal washing medium (Hanks' balanced salt solution containing 0·1% bovine serum albumin) or spent supernatant from P3NS1 cultures. The cells were washed ×3 and incubated for a further 1 h on ice with Protein A (Pharmacia) labelled with $^{125}$I by the method of Williams *et al*. (1977). The amount of protein used was 5 ng per well, giving between $2 \times 10^4$ and $10^5$ ct/min/well, according to the batch used. The cells were washed ×6 and dried down. After spraying with a plastic film (Nobecutane) the wells were separated with a band saw and the bound $^{125}$I measured in a gamma counter.

*Absorption*.—Hybridoma supernatant was diluted 1:10 and absorbed in some tests with cultured tumour cells at a concentration of $10^8$ cells per ml of supernatant for 2 h at ambient temperature. The cells were removed by centrifugation and the absorbed supernatant tested for reactivity against selected targets (see text).

### RESULTS

One fusion produced 48 growing hybrid cultures from 48 explants. All were screened for reactivity against 791T target cells and 5 were found initially to react significantly above control levels. Upon repeated testing only two (designated 791T/36 and 791T/48) were consistently positive, and these were further tested for reactivity against several other target cell lines (Figure). Both were positive for 791T and a second osteogenic sarcoma cell line, 788T, and the 791T/36 supernatant was also positive for a third osteogenic sarcoma, 805T. Reactivity of 791T/36 against a fourth sarcoma, 888T, was raised but not significantly different from background. All other tests were negative, and this includes 3 fibroblastic lines derived from the donor of the 791T cell line (860, 870 and 791SK, respectively). At this point the 791T/36 hybridoma was cloned in soft agar.

Twelve clones were isolated and their supernatants tested against 791T cells, 791SK skin fibroblasts and two other cell lines (618 Lu normal lung cells and RAJ1 lymphoma cells). Nine clones were highly reactive with 791T cells and 3 more weakly so, but all were negative with the other target cells (Table I). Three of the strongly reactive clones were screened against an extensive panel of target cells, as shown in Table II. All 3 clones had similar activity, presumably indicating that the 791/36 hybridoma culture might contain only one positive clone. They gave positive antibody reactions against 4/10 osteogenic sarcomas from individual patients, but were completely negative with fibroblastic cells, whether derived from the donor of 791T or from other patients. Two of the allogeneic fibroblast controls (788SK and

FIGURE.—Specificity of anti-791T hybridoma supernatants. The supernatants from hybridoma cultures 791T/36 and 791T/48 were tested against various target cells to test their specificity before cloning. The background (ct/min with P3NS1 medium) was 295 ± 35, and is converted to zero in the figure, *i.e.* the histograms are corrected for background by subtraction. The vertical bars on the histograms represent s.e.

TABLE I.—*Reactivity of clones of Hybridoma* 791*T*/36 *against* 791*T osteogenic sarcoma and control cell lines*

| Test supernatant | $^{125}$I (ct/min ± s.d.) bound to target cells: Osteogenic sarcoma 791T* | Skin fibroblasts 791SK* | Lung fibroblasts 618 Lu | Burkitt lymphoma Raji |
|---|---|---|---|---|
| HBSS + BSA† | 278 ± 4 | 689 ± 15 | 396 ± 36 | 694 ± 31 |
| P3NS1 spent medium | 279 ± 50 | 410 ± 55 | 366 ± 6 | 485 ± 96 |
| 791T/36 Clone 1 | 12082 ± 402 | 326 ± 30 | 575 ± 52 | 719 ± 29 |
| Clone 2 | 14932 ± 598 | 380 ± 21 | 549 ± 22 | 522 ± 114 |
| Clone 3 | 16905 ± 261 | 404 ± 34 | 447 ± 6 | 444 ± 71 |
| Clone 4 | 16311 ± 322 | 316 ± 40 | 433 ± 33 | 473 ± 164 |
| Clone 5 | 10700 ± 388 | 339 ± 29 | 318 ± 8 | 591 ± 41 |
| Clone 6 | 13981 ± 371 | 361 ± 11 | 471 ± 9 | 632 ± 132 |
| Clone 7 | 921 ± 77 | 292 ± 14 | 307 ± 63 | 259 ± 36 |
| Clone 8 | 1174 ± 44 | 501 ± 51 | 369 ± 44 | 329 ± 36 |
| Clone 9 | 1858 ± 173 | 316 ± 16 | 455 ± 43 | 226 ± 24 |
| Clone 10 | 18383 ± 1236 | 334 ± 24 | 488 ± 19 | 229 ± 20 |
| Clone 11 | 18018 ± 63 | 657 ± 57 | 608 ± 118 | 276 ± 25 |
| Clone 12 | 11189 ± 497 | 425 ± 25 | 435 ± 57 | 323 ± 97 |

* 791T and 7915K were derived from the same patient.
† Hanks' blanced salt solution + 0·1% bovine serum albumin (washing medium).

805SK) were from donors of sarcomas that reacted positively (788T and 805T, respectively). Erythrocytes from 9 control donors (comprising all major blood groups) and their peripheral mononuclear cells were all negative, as were sheep erythrocytes, a canine osteogenic sarcoma (73-2295) and rat tumour cell lines. Tests against 19 other human tumour cultures of various types, however, revealed cross-reactions against HeLa cells, EB33 prostate carcinoma, HT29 colon carcinoma and A549 lung carcinoma cells. These cross-reactions, and all the negative reactions, were always reproducible upon repeated testing.

*Absorption tests*

Supernatant from 791T/36 Clone 3 was diluted and absorbed for 2 h with various cell lines at a concentration of $10^8$ cells/ml.

TABLE II.—*Reactivity of certain 791T/36 clones against various target cells*

| | | Binding ratio† of 791T/36 clone | | |
|---|---|---|---|---|
| Cell line | Cell type | 3 | 4 | 10 |
| 791T‡ | Osteogenic sarcoma | 22·84*** | 25·60*** | 22·84*** |
| 788T§ | ,, ,, | 46·82*** | 53·13*** | 52·74*** |
| 845T | ,, ,, | 3·19* | 5·58** | NT¶ |
| 805T‖ | ,, ,, | 2·63 | 3·41* | 2·99* |
| 803T | ,, ,, | 1·45 | 1·45 | NT |
| 836T | ,, ,, | 0·78 | 0·73 | 0·82 |
| 706T | ,, ,, | 1·89 | 2·49 | NT |
| 781T | ,, ,, | 1·83 | 2·06 | 1·92 |
| 888T | ,, ,, | 1·08 | 1·13 | 1·12 |
| 792T | ,, ,, | 1·12 | 1·23 | NT |
| 791SK‡ | Skin fibroblasts | 0·99 | 0·77 | 0·84 |
| 788SK§ | ,, ,, | 0·90 | NT | 0·99 |
| 805SK‖ | ,, ,, | 1·41 | 2·05 | 1·46 |
| 181SK | ,, ,, | 2·14 | 2·18 | 2·10 |
| 836SK | ,, ,, | 1·31 | 1·29 | NT |
| 860‡ | Tumour-derived fibroblasts | 1·39 | NT | 1·48 |
| 870‡ | ,, ,, | 1·09 | 1·04 | 0·95 |
| 618Lu | Lung fibroblasts | 1·68 | 1·63 | 1·83 |
| 74BM | Foetal marrow | 1·50 | 1·69 | NT |
| — | Human erythrocytes | 0·61–0·91 | 0·61–1·35 | NT |
| — | Human mononuclear cells | 0·61–1·24 | 0·70–2·00 | 0·63–1·28 |
| HT29 | Colon carcinoma | 6·66** | 7·10** | 6·85** |
| HTC8 | ,, ,, | 1·61 | 2·52 | 1·89 |
| HRT18 | ,, ,, | 1·21 | 1·02 | 1·10 |
| 734 B | Breast carcinoma | 2·18 | 1·87 | 1·81 |
| SK Br 3 | ,, ,, | 1·54 | 1·38 | 1·47 |
| HS 578T | ,, ,, | 1·22 | 1·76 | 1·32 |
| MeWo | Melanoma | 0·98 | 0·72 | 0·88 |
| Mel 57 | ,, | 1·19 | 1·25 | 1·22 |
| Mel 2a | ,, | 1·36 | 2·17 | 2·07 |
| NK1-4 | ,, | 1·25 | 0·47 | 0·56 |
| RPM1 5966 | ,, | 1·36 | 1·48 | 1·66 |
| A549 | Lung carcinoma | 4·54** | 5·93** | 4·76** |
| A427 | ,, ,, | 1·09 | 1·31 | 1·10 |
| 9812 | ,, ,, | 1·39 | 1·22 | 0·94 |
| HeLa | Cervix carcinoma | 55·52*** | 53·95*** | 53·58** |
| EB33 | Prostate carcinoma | 28·3*** | 26·11*** | 26·22*** |
| T24 | Bladder carcinoma | 1·50 | 1·76 | 1·48 |
| PA-1 | Ovarian carcinoma | 1·61 | 1·50 | 1·43 |
| RAJ1 | Burkitt lymphoma | 1·26 | 1·06 | 0·60 |
| — | Sheep erythrocytes | 1·00 | 0·56 | 0·57 |
| 73-2295 | Canine osteosarcoma | 1·10 | 1·43 | 1·34 |
| D23 | Rat hepatoma | 0·78 | 0·98 | 0·88 |
| KXD2 | ,, ,, | 1·01 | 1·12 | 1·10 |
| Sp4 | Rat breast carcinoma | 1·25 | 1·15 | 1·27 |

† Binding ratio = mean ct/min with 791T/36 clone supernatant, divided by mean ct/min with P3NS1 spent medium. P3NS1 spent medium gave the same ct/min as washing medium. Statistical analysis of the difference between ct/min for 791T/36 clone and ct/min for P3NS1 medium by the *t* test is indicated by: ***($P < 0{\cdot}001$); **($P < 0{\cdot}01$) and *($P < 0{\cdot}05$).

‡ 791T, 791SK, 860 and 870 were from the same patient (M.U.).

§ 788T and 788SK were from the same patient (P.R.).

‖ 805T and 805SK were from the same patient (Q.L.).

¶ NT = Not tested.

TABLE III.—*Absorption of antibody from 791T/36 Clone 3 supernatant*

| Target cells | Absorbing cells† | Mean ct/min—Background‡ unabsorbed | absorbed§ | Reduction on absorption (%) |
|---|---|---|---|---|
| 791T | 791T | 3771‖ | 107 | 97·2 |
| 791T | 788T | 3771 | 296 | 92·1 |
| 791T | EB33 | 3771 | 182 | 95·2 |
| 791T | Mel2a* | 20622‖ | 15652 | 24·1 |
| 791T | Mel57* | 20622 | 17499 | 15·1 |
| 791T | PA-1* | 20622 | 18339 | 11·1 |
| 791T | A549 | 20622 | −798 | 100 |
| 788T | 788T | 4271 | 366 | 91·4 |
| 788T | 791T | 4271 | 296 | 93·1 |
| A549 | A549 | 6053 | 1481 | 75·5 |
| A549 | 791T | 6053 | 235 | 96·1 |
| HT29 | HT29 | 1697 | 512 | 69·8 |
| HT29 | 791T | 1697 | 412 | 75·7 |
| EB33 | EB33 | 5846 | 322 | 94·5 |
| EB33 | 791T | 5846 | 323 | 94·5 |
| HeLa | HeLa | 2330 | 180 | 92·3 |
| HeLa | 791T | 2330 | 240 | 89·7 |

† Cells marked * did not cross-react with the antibody in direct tests; all other cells in this Table did so (see Table II).

‡ Ct/min with P3NS1 spent medium.

§ Supernatant 791T/36 Clone 3 was diluted 1:10 and absorbed with $10^8$ cells/ml at room temperature for 2 h.

‖ The two indicated values were obtained in separate experiments with different batches (and $^{125}$I input levels) of labelled Protein A.

These conditions were based upon those used successfully with a monoclonal antibody to a rat breast carcinoma, which has a titre against relevant target cells of 1/1000 (Gunn *et al.*, 1980); this was also the titre empirically determined for 791T/36 Clone 3. Table III shows data from experiments in which the monoclonal antibody was (a) absorbed by 791T cells and tested against other cross-reactive target cells, (b) absorbed with other tumour cells and tested against 791T, or (c) absorbed with, and tested against cells other than 791T. Where the cross-tested absorbing cells or target cells were reactive with the antibody (Table II) reductions of bound ct/min of between 70% and 90% were obtained after absorption. When the antibody was absorbed with non-cross-reactive cells (Mel 2a, Mel 57, PA-1) however, the reduction of activity against 791T cells was only 11–24%. These results support the specificity of the antibody demonstrable in direct tests.

### DISCUSSION

Monoclonal antibodies were obtained from the cloned hybridoma 791T/36, which reacted preferentially with tumour cells. The antibody was strongly reactive with the immunizing osteogenic–sarcoma cell line, 791T, but was negative with fibroblasts obtained from the same patient (791SK, 860 and 870). It was also strongly reactive with a second osteogenic sarcoma, 788T, and more weakly reactive with another, 805T, but was negative with skin fibroblasts from the same patients (788SK and 805SK, respectively). This clearly demonstrates that the antibody was not reacting against human species-associated or histocompatibility antigens on the sarcoma cells. The antibody also failed to react with 25 other cultured human target cell lines, so was not directed against FCS components which are sometimes incorporated into the membrane of cells grown in medium containing this as a supplement (Embleton & Iype, 1978; Irie *et al.*, 1974). Red blood cells from volunteer donors, including all the major blood groups, did not react with the antibody, so it can not have been directed against blood-group antigens, which are sometimes expressed on tumour cells. Other antigens which can be discounted are DR antigens and Forssman antigen, since no reaction was obtained with peripheral mononuclear cells (containing both lymphocytes and monocytes) from 9 different donors, or with sheep red blood cells, respectively. It thus seems certain that the antibody was directed against a tumour-associated antigen.

However, the antigen detected was not shared by all the osteogenic sarcomas, since only 4/10 were positive, and was not exclusive to osteogenic sarcomas. Positive (and reproducible) cross-reactions were obtained with one cell line each derived from carcinomas of colon, lung, prostate and cervix. This does not represent a tissue-related cross-reactivity, because 2 other colon carcinomas and 2 lung carcinoma cell lines were negative. Rather, the picture is one of a randomly expressed tumour-associated antigen not associated with any particular histological type of tumour.

This is at variance with the dogmatic view that human tumours express antigens common to tumours of a given tissue (discussed by Baldwin & Embleton, 1977), which view appears to be supported by some of the early studies on monoclonal antibodies to human tumours, in which tissue-related specificity was reported (Koprowski *et al.*, 1978; Steplewski *et al.*, 1979; Yeh *et al.*, 1979; Koprowski *et al.*, 1979; Carrel *et al.*, 1980). However, preliminary results with clones derived from the second anti-791T hybridoma, 791T/48, confirm the present findings of random antigen expression (Embleton *et al.*, to be published), and further support is provided by cross-reactions we have observed with anti-melanoma monoclonal antibodies (unpublished). Monoclonal antibodies potentially offer the best means of analysing the antigenic profiles of human solid tumours, and we suggest that the emergent picture may well be one of a spectrum of antigens, one or several of which may be expressed by any given tumour irrespective of histological type, perhaps in addition to differentiation antigens which might be tissue-related. If this eventually proves to be a general rule, attempts to use monoclonal antibodies for monitoring or therapy of human tumours may have to depend upon the use of panels of monoclonal antibodies capable of recognizing many different antigens.

This work was supported by the Cancer Research Campaign, London, U.K. We thank Mrs W. O. Ward for technical assistance. Cell lines were obtained from Dr C. Sorg, Dr P. Burtin, Dr J. De Vries, Dr M. Moore, Professor F. Schöder, Professor M. A. Epstein, and the U.S. Naval Biomedical Center, Oakland, California, by arrangement with Dr W. A. Nelson-Rees.

### REFERENCES

AL-SHEIKLY, A. W. A. R., EMBLETON, M. J. & PRICE, M. R. (1980) Detection of tumor specific antigens and alloantigens using a radioisotopic antiglobulin assay. In *Biology of the Cancer Cell.* Ed. Letnansky. Amsterdam: Kugler. p. 121.

BALDWIN, R. W. & EMBLETON, M. J. (1977) Assessment of cell-mediated immunity to human tumor-associated antigens. *Int. Rev. Exp. Pathol.*, **17**, 49.

CARREL, S., ACCOLLA, R. S., CARMAGNOLA, A. L. & MACH, J.-P. (1980) Common human melanoma-associated antigen(s) detected by monoclonal antibodies. *Cancer Res.*, **40**, 2523.

EMBLETON, M. J. & IYPE, P. T. (1978) Surface antigens of rat liver epithelial cells grown in medium containing foetal bovine serum. *Br. J. Cancer*, **38**, 456.

GALFRE, G., HOWE, S. C., MILSTEIN, C., BUTCHER, G. W. & HOWARD, J. C. (1977) Antibodies to major histocompatibility antigens produced by hybrid cell lines. *Nature*, **266**, 550.

GUNN, B., EMBLETON, M. J., MIDDLE, J. G. & BALDWIN, R. W. (1980) Monoclonal antibody against a naturally occurring rat mammary carcinoma. *Int. J. Cancer*, **26**, 325.

HOLLINSHEAD, A. C., STEWART, T. H. M. & HERBERMAN, R. B. (1974) Delayed-hypersensitivity reactions to soluble membrane antigens of human malignant lung cells. *J. Natl Cancer Inst.*, **52**, 327.

IRIE, R. F., IRIE, K. & MORTON, D. L. (1974) Natural antibody in human serum to a neoantigen in human cultured cells growing in foetal bovine serum. *J. Natl Cancer Inst.*, **52**, 1051.

KENNET, R. H. & GILBERT, F. (1979) Myeloma producing antibodies against a human neuroblastoma antigen present on foetal brain. *Science*, **203**, 1120.

KOPROWSKI, H., STEPLEWSKI, Z. & HERLYN, D. (1978) Study of antibodies against human melanoma produced by somatic cell hybrids. *Proc. Natl Acad. Sci.*, **75**, 3405.

KOPROWSKI, H., STEPLESWKI, Z., MITCHELL, K., HERLYN, D., HERLYN, M. & FUHRER, P. (1979) Colorectal-carcinoma antigens detected by hybridoma antibodies. *Somatic Cell Gen.*, **5**, 957.

STEPLEWSKI, Z., HERLYN, D., CLARK, W. H. & KOPROWSKI, H. (1979) Reactivity of monoclonal antibodies with melanoma cells freshly isolated from primary and metastatic melanoma. *Eur. J. Immunol.*, **9**, 94.

WILLIAMS, A. F., GALFRE, G. & MILSTEIN, C. (1977) Analysis of cell surfaces by xenogeneic myeloma-hybrid antibodies: Differentiation antigens of rat lymphocytes. *Cell*, **12**, 663.

YEH, M.-Y., HELLSTRÖM, I., BROWN, J. P., WARNER, G. A., HANSEN, J. A. & HELLSTRÖM, K. E. (1979) Cell surface antigens of human melanoma identified by monoclonal antibody. *Proc. Natl Acad. Sci.*, **76**, 2927.